# Aronia Berry Extract Modulates MYD88/NF-kB/P-Glycoprotein Axis to Overcome Gemcitabine Resistance in Pancreatic Cancer

**DOI:** 10.3390/ph17070911

**Published:** 2024-07-09

**Authors:** Yuan Li, Caiming Xu, Haiyong Han, Silvia Pascual-Sabater, Cristina Fillat, Ajay Goel

**Affiliations:** 1 Department of Molecular Diagnostics and Experimental Therapeutics, Beckman Research Institute of City of Hope, Biomedical Research Center, Monrovia, CA 91016, USA; yuali@coh.org (Y.L.); caixu@coh.org (C.X.); 2 Department of Clinical Laboratory, Yangpu Hospital, Tongji University School of Medicine, Shanghai 200090, China; 3 Department of General Surgery, The First Affiliated Hospital of Dalian Medical University, Dalian 116004, China; 4 Division of Molecular Medicine, The Translational Genomics Research Institute, Phoenix, AZ 85004, USA; hhan@tgen.org; 5 Institut d’Investigacions Biomèdiques August Pi i Sunyer (IDIBAPS), 08036 Barcelona, Spain; sipascual@clinic.cat (S.P.-S.); cfillat@clinic.cat (C.F.); 6 City of Hope Comprehensive Cancer Center, Duarte, CA 91010, USA

**Keywords:** pancreatic ductal adenocarcinoma, aronia berry extracts, gemcitabine resistance, MYD88, anticancer effect, chemoprevention

## Abstract

Pancreatic ductal adenocarcinoma (PDAC) is a highly lethal disease with poor survival rates, primarily due to the limited effectiveness of gemcitabine (Gem)-based chemotherapy, as well as the acquisition of chemotherapeutic resistance. Aronia berry extracts (ABEs), abundant in phenolic constituents, have been recently recognized for their anticancer properties as well as their encouraging potential to help overcome chemoresistance in various cancers. In the present study, we explored ABE’s potential to overcome Gem resistance in PDAC and identify specific growth regulatory pathways responsible for its anticancer activity. Through a series of in vitro experiments in gemcitabine-resistant (Gem-R) cells, we elucidated the synergistic interactions between Gem and ABE treatments. Using advanced transcriptomic analysis and network pharmacology, we revealed key molecular pathways linked to chemoresistance and potential therapeutic targets of ABE in Gem-R PDAC cells. Subsequently, the findings from cell culture studies were validated in patient-derived 3D tumor organoids (PDOs). The combination treatment of ABE and Gem demonstrated significant synergism and anticancer effects on cell viability, proliferation, migration, and invasion in Gem-R cells. Transcriptomic analysis revealed a correlation between the NF-Κb signaling pathway and Gem-R (*p* < 0.05), exhibiting a marked upregulation of MYD88. Additionally, MYD88 exhibited a significant correlation with the overall survival rates in patients with PDAC patients in the TCGA cohort (HR = 1.58, *p* < 0.05). The MYD88/NF-Κb pathway contributes to chemoresistance by potentially upregulating efflux transporters like P-glycoprotein (P-gp). Our findings revealed that the combined treatment with ABE suppressed the NF-Κb pathway by targeting MYD88 and reducing P-gp expression to overcome Gem resistance. Lastly, the combination therapy proved highly effective in PDOs in reducing both their number and size (*p* < 0.05). Our study offers previously unrecognized insights into the ability of ABE to overcome Gem resistance in PDAC cells through its targeting of the MYD88/NF-κb/P-gp axis, hence providing a safe and cost-effective adjunctive therapeutic strategy to improve treatment outcomes in PDAC.

## 1. Introduction

Pancreatic ductal adenocarcinoma (PDAC) is the third leading cause of cancer-related deaths. Despite the 5-year overall survival (OS) rates in the United States having improved from less than 5% in 1990 to as high as ~10% in 2023, the prognosis for patients with PDAC remains distressingly low. This high rate of mortality is primarily attributed to the difficulty in early diagnosis of pancreatic cancer, as well as the inadequate efficacy of the currently available treatment options [1,2]. Gemcitabine (Gem)-based chemotherapy has played a crucial role in advancing the treatment of patients with PDAC since its initial endorsement as a primary therapeutic option in 1997. Nab-paclitaxel combined with Gem hydrochloride is a commonly used therapeutic regimen in PDAC [3,4,5,6,7]. However, its efficacy is notably limited by the emergence of drug resistance in most patients, highlighting a primary challenge for the effective management of this lethal malignancy [8,9]. The newer combination therapeutic regimens, such as FOLFIRINOX, while resulting in slightly improved treatment outcomes, exhibit significant toxicity and are expensive. Collectively, these findings highlight the critical importance of gaining deeper insights into the molecular mechanisms underlying Gem resistance and investigating novel therapeutic approaches to overcome chemoresistance in PDAC and improve patient outcomes.

Previous studies have demonstrated the pivotal role of ABC drug transporters in conferring therapeutic resistance to Gem in PDAC [10,11]. Among these, P-glycoprotein (P-gp) is the most recognized and established regulator contributing to chemoresistance [12]. Also known as ABCB1 or MDR1, P-gp plays a vital role in the emergence of multidrug resistance (MDR) and was the first human ABC transporter to be characterized [13]. P-gp has been shown to affect the uptake, distribution, and elimination of compounds through modulation of cellular efflux function [14]. Furthermore, the efficacy of several chemotherapeutic agents, including paclitaxel (PTX), cisplatin (CP), and doxorubicin (DOX), can be affected by P-gp overexpression. However, P-gp inhibitors such as quinidine and verapamil have been plagued by their toxicity and poor selectivity, in turn limiting their clinical use [14,15,16]. Thus, exploring safer and more effective P-gp inhibitors remains highly valuable in various cancers.

More recently, there is growing enthusiasm to explore the possibility of using various natural products and their derivatives as complementary and alternative therapeutic approaches, given their multi-targeted efficacy and safety in various cancers [17,18,19,20,21,22,23,24,25,26,27,28,29]. These natural compounds include curcumin [22,30,31,32,33,34,35,36], Andrographis [29,37,38,39], resveratrol [40,41], and ginseng [42], all of which have been studied for their adjunctive and synergistic efficacy in various cancers, including PDAC. Additionally, several of these natural remedies have been identified as inhibitors of P-gp, MRP1, MRP2, and BCRP and can thus sensitize cancer cells to various chemotherapeutic drugs. A range of natural compounds such as flavonoids, coumarins, resins, saponins, and terpenoids have been explored for their ability to combat drug resistance in cancer by inhibiting P-gp [43]. In particular, aronia berries (black chokeberries) are thought to have anticancer potential due to their richness in phenolic compounds that possess potent antioxidant and anti-inflammatory properties, such as procyanidins, anthocyanins, phenolic acids, and their analogs [44]. Previous studies also indicated that derivatives of aronia berry extract (ABE) can impede the formation of breast cancer stem cells, making it a promising candidate for cancer chemoprevention [45]. Furthermore, *Aronia Melanocarpa* extract has been reported to impede the growth of PDAC through induction of apoptosis in pancreatic cancer cell lines, such as AsPC-1 [46]. While recent research has suggested the anticancer effects of ABE in PDAC, its specific impact on chemosensitivity, especially when combined with chemotherapeutic agents, is largely unexplored. Given that most natural medicines are used as adjuncts to conventional chemotherapy, we hypothesized that ABE might have the potential to inhibit tumor growth and overcome chemoresistance in PDAC.

In our current study, we conducted a comprehensive set of experiments in PDAC cell lines and patient-derived 3D organoids (PDOs) to gain additional insights into the molecular mechanisms of ABE’s anticancer effects and its potential in overcoming Gem resistance in PDAC. 

## 2. Results

### 2.1. The Treatment of ABE and Gemcitabine Shows Synergistic Inhibition in Gem-R Pancreatic Cancer Cells 

**Figure 1:**
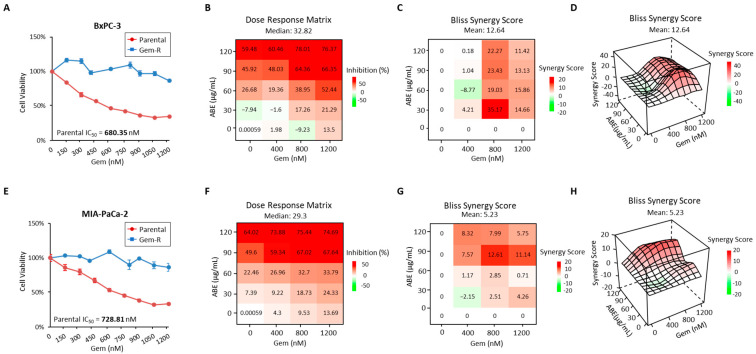
Identification of drug resistance in Gem-R PDAC cells and the treatment ABE with Gem synergistically inhibits the growth of Gem-R PDAC cells. The IC_50_ of parental BxPC-3/Gem-R BxPC-3 (**A**) and parental MIA-PaCa-2/Gem-R MIA-PaCa-2 (**E**) to Gem were calculated using the CCK8 assay. Error bars are the mean ± SD. Percentage of growth inhibition of Gem-R BxPC-3 cell lines (**B**) and Gem-R MIA-PaCa-2 (**F**) cell lines treated in multiple combinations of ABE (0, 30, 60, 90, and 120 μg/mL) and Gem (0, 400, 800, and 1200 nM). The dose–response matrix was measured using the CCK8 assay. Bliss synergy values were calculated from data in B and F, accessed as a 2D-contour (**C**,**G**) and 3D-contour (**D**,**H**) drug dose–response model evaluating cell viability after treatment with ABE in Gem-R PDAC cells.

In our present investigation, we initially utilized the two Gem-resistant (Gem-R) cell lines as described in our prior study [34]. Notably, the half-maximal inhibitory concentration (IC_50_) of Gem was substantially higher in Gem-R BxPC-3 and Gem-R MIA-PaCa-2 cells compared to their respective parental cell lines (Figure 1A,E). Specifically, the IC_50_ of Gem in the parental cells was determined to be 680.35 nM and 728.81 nM, respectively. These values were significantly lower than those observed in the Gem-R counterparts, Gem-R BxPC-3 and Gem-R MIA-PaCa-2, which exhibited IC_50_ values exceeding 1200 nM. Next, we determined that the ABE could reduce Gem resistance in Gem-R PDAC cells. Subsequently, we administered concurrent treatments with varying concentrations of ABE (0, 30, 60, 90, and 120 μg/mL) and Gem (0, 400, 800, and 1200 nM), demonstrating a synergistic reduction in cell viability. Next, we investigated the anticancer effects of ABE on Gem-R BxPC-3 and Gem-R MIA-PaCa-2 cell lines. These resistant cell lines were treated with ABE for 48 h, and cell viability was assessed using the CCK-8 assay. The results demonstrated a dose-dependent inhibition of cell viability by ABE, with IC_50_ values of 110.97 μg/mL for Gem-R BxPC-3 and 89.17 μg/mL for Gem-R MIA-PaCa-2 (Supplementary Figure S1). Ultimately, the criteria for the optimal dosage ratio of ABE and Gem in the combination treatment was established by both dose–response curves needing to achieve over 50% inhibition and the Bliss synergy score exceeding 10 points. In these treatments, the ABE/Gem ratio of 90:800 exhibited a synergistic anticancer effect in both cell lines. In the Gem-R BxPC-3 cell line, under this ratio, the inhibitory rate was 64.36%, in contrast to −9.23% with only Gem treatment and 45.92% with only ABE treatment, resulting in a synergy score of 23.43 (Figure 1B–D). Similarly, in the Gem-R MIA-PaCa-2 cell line, under the same ratio, the inhibitory rate was 67.02%, compared to 9.53% with only Gem treatment and 49.6% with only ABE treatment, yielding a synergy score of 12.61 (Figure 1F–H). Hence, all subsequent experiments were conducted using a concentration of 90 μg/mL ABE and 800 nM Gem.

### 2.2. The Combination of ABE and Gemcitabine Inhibits Cell Proliferation, Colony Formation, Migration, and Invasion in Gem-R PDAC Cell Lines

**Figure 2:**
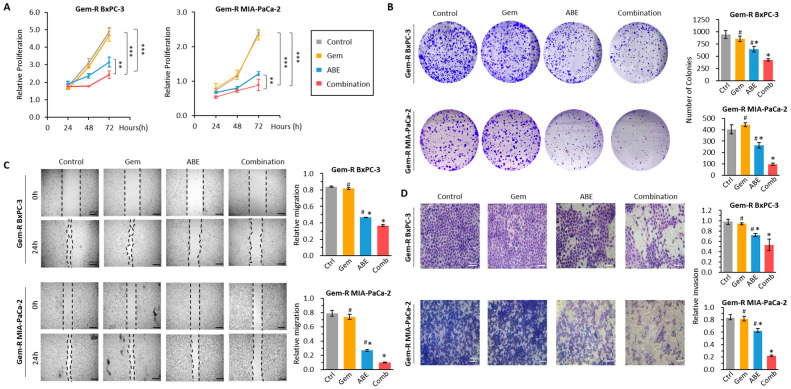
Aronia berry extract exhibits anti-proliferation, migration, and invasion effects in Gem-R PDAC cells. (**A**) Cell proliferation was compared in Gem-R cell lines treated with Gem, ABE, and their combination. Cell viability was assessed using a CCK-8 assay at 24, 48, and 72 h time points. (**B**) A colony formation assay was conducted to evaluate the clonogenicity of each group after a 48 h exposure to the treatments. Representative images of colonies were taken, and the number of colonies was counted. (**C**) A wound healing assay was performed on Gem-R PDAC cells treated with Gem, ABE, and their combination for 48 h. Representative images of the wound and the recovering areas (marked by black lines) were taken, and the percentage of wound closure was measured. (**D**) A transwell assay was carried out on Gem-R PDAC cells treated with Gem, ABE, and their combination for 48 h. The number of invading cells was counted in three randomly selected fields on the membrane. * *p* indicates *p* < 0.05 vs. control group; # *p* indicates *p* < 0.05 vs. combination group. ** *p* indicates *p* < 0.01; *** *p* indicates *p* < 0.001.

To evaluate the potential effects of ABE in enhancing chemosensitivity to Gem in Gem-R PDAC cells, we conducted a series of functional experiments, including colony formation, wound healing (scratch), and invasion assays. To evaluate the impact of the combination of ABE and Gem on PDAC cell proliferation, we treated Gem-R BxPC-3 and Gem-R MIA-PaCa-2 cells with Gem (800 nM) and ABE (90 µg/mL), either separately or in combination for 48 h. We performed CCK-8 assays, which revealed that the combination of Gem and ABE significantly outperformed the individual treatments (*p* < 0.01 vs. Gem; *p* < 0.01 vs. ABE in Gem-R MIA-PaCa-2 cells; *p* < 0.01 vs. Gem; *p* < 0.01 vs. ABE in Gem-R BxPC-3 cells; Figure 2A). Furthermore, the colony formation assay demonstrated a significant reduction in clonogenicity with the combination of Gem and ABE compared to individual treatments in BxPC-3 cells (combination vs. Gem: fold change [FC] = 0.49, *p* < 0.01; combination vs. ABE: FC = 0.66, *p* < 0.01, Figure 2B) and MIA-PaCa-2 cell lines (combination vs. Gem: FC = 0.22, *p* < 0.01; combination vs. ABE: FC = 0.37, *p* < 0.01, Figure 2B). To determine whether the combination of Gem and ABE had a more significant impact on the motility and invasive potential of PDAC cells, we conducted scratch–wound and transwell assays. The scratch–wound assay revealed that the combination of Gem and ABE significantly inhibited cell migration to a greater extent than individual treatments in both BxPC-3 (combination vs. Gem: FC = 0.22, *p* < 0.01; combination vs. ABE: FC = 0.37, *p* < 0.01, Figure 2C) and MIA-PaCa-2 cell lines (combination vs. Gem: FC = 0.14, *p* < 0.01; combination vs. ABE: FC = 0.37, *p* < 0.01, Figure 2C). The transwell assay showed that a combination of Gem and ABE shows a significantly more significant reduction in invasion than individual treatment in both BxPC-3 (combination vs. Gem: FC = 0.53, *p* < 0.01; combination vs. ABE: FC = 0.68, *p* < 0.01, Figure 2D) and MIA-PaCa-2 cell lines (combination vs. Gem: FC = 0.20, *p* < 0.01; combination vs. ABE: FC = 0.27, *p* < 0.01, Figure 2D). Furthermore, epithelial-to-mesenchymal transition (EMT) and matrix metalloproteinase-related proteins are closely linked to tumor metastasis and invasion. We examined the expression of these proteins following treatments with gemcitabine, ABE, and their combination. Our findings revealed that the combination treatment significantly reduced the expression of proteins associated with migration and metastasis (e.g., MMP9, vimentin) while significantly increasing the expression of those that promote cell adhesion (e.g., E-cadherin; Supplementary Figure S2).

Consistent with the previous results, our findings demonstrated that the combination of Gem and ABE significantly reduced the migration and invasion abilities of PDAC cells.

### 2.3. ABE, in Combination with Gemcitabine, Promotes Cell Apoptosis

**Figure 3:**
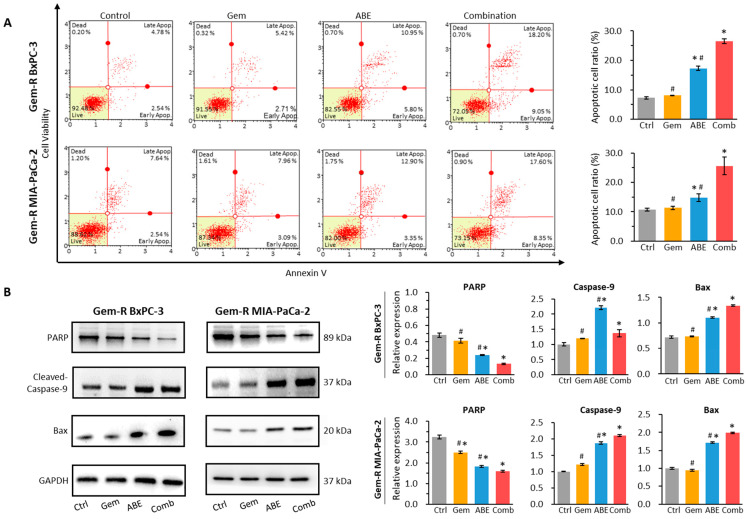
Aronia berry extract induces cell apoptosis in Gem-R PDAC cells. (**A**) Annexin V assay was conducted to measure the percentage of apoptotic cells on Gem-R PDAC cells treated with Gem, ABE, and their combination for 48 h. (**B**) Western blot analysis was performed on Gem-R PDAC cells treated with Gem, ABE, and their combination for 48 h. The relative levels of PARP, caspase-9, and Bax were quantitatively analyzed by comparing them to the control GAPDH expression. * *p* indicates *p* < 0.05 vs. control group; # *p* indicates *p* < 0.05 vs. combination group.

To evaluate the apoptotic effects of the combination of Gem and ABE on PDAC cells, we initially conducted an annexin V binding assay. The analysis indicated that the combination of Gem and ABE increased the rate of apoptosis in both Gem-R cell lines (Figure 3A) compared to the individual administrations of Gem and ABE (BxPC-3 cell line: combination vs. Gem: 26.55% vs. 8.095%, *p* < 0.01; combination vs. ABE: 26.55% vs. 17.33%, *p* < 0.01, Figure 3A; and MIA-PaCa-2 cell line: combination vs. ABE: 25.60% vs. 11.29%, *p* < 0.01; combination vs. ABE: 25.60% vs. 14.8%, *p* < 0.01, Figure 3A). Next, we conducted Western blotting (WB) to analyze changes in apoptotic proteins following treatment. The WB analysis demonstrated that the combination of Gem and ABE increased the expression of cleaved caspase-9 and Bax, pro-apoptotic proteins, while simultaneously reducing the expression of total PARP, an anti-apoptotic protein, in comparison to the control and individual treatments (Figure 3B) [47,48,49,50,51]. Collectively, these data confirm that the combination of Gem and ABE induced more Gem-R PDAC cell apoptosis compared to individual treatments in vitro.

### 2.4. The MYD88/NF-κB Signaling Pathway Is Aviated in Gem-R PDAC Cells

**Figure 4:**
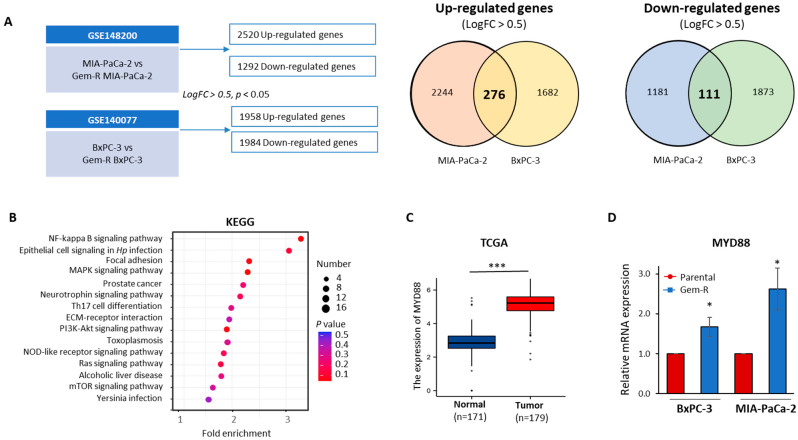
The MYD88/NF-κB signaling pathway is associated with Gem resistance in PDAC cells. (**A**) A schematic illustrates the differentially expressed genes in the BxPC-3-R vs. BxPC-3-Parental cell line (GSE 140077) and MIA-PaCa-2-R vs. MIA-PaCa-2-Parental cell line (GSE 148200). The Venn diagram represents the upregulated and downregulated expression of genes selected using Log2FC > 0.5 and *p* < 0.05. (**B**) Scatter plots of KEGG pathway enrichment analysis of up- and downregulated genes in Gem-R PDAC cell lines. The top 15 significantly enriched KEGG pathways were prioritized. The circle represents the number of differentially expressed genes, while the circle color represents the range of *p*-values. (**C**) The expression level of the selected genes, MYD88, was analyzed using PDAC cases from the TCGA dataset. (**D**) qRT-PCR assays were undertaken to measure the mRNA expression levels of selected genes in Gem-R and parental PDAC cell lines, with *β-actin* expression as the internal control. * *p* indicates *p* < 0.05 vs. control group; *** *p* indicates *p* < 0.001.

To investigate the mechanism of Gem resistance in PDAC cells, we conducted gene expression profiling analysis by comparing parental and Gem-R PDAC cells using publicly available Gene Expression Omnibus (GEO) datasets (GSE148200 and GSE140077). We identified 2520 upregulated and 1292 downregulated genes in MIA-PaCa-2 cells, and 1958 upregulated and 1984 downregulated genes in the BxPC-3 cell line. These genes were selected based on specific criteria: a log2FC > ±0.5 and a *p*-value < 0.05. (Figure 4A). Subsequently, we focused on the genes that were consistently regulated in the same direction in both cell lines, identifying 276 upregulated and 111 downregulated genes. We performed KEGG pathway analysis to gain further insights into the biological pathways associated with these significantly dysregulated genes. This analysis was carried out utilizing the DAVID database (https://david.ncifcrf.gov/, accessed on 28 August 2022) [52,53]. The top 15 enrichment pathways were prioritized (Figure 4B). Among these, of particular interest was the MYD88/NF-κB signaling pathway, which ranked as the top pathway and has been the subject of numerous previous studies highlighting its potential involvement in PDAC and Gem resistance [54,55,56,57]. Additionally, previous research has shown that MYD88 expression is increased in cancer tissues and correlated with paclitaxel resistance in other cancers such as breast, ovarian, and lung [58,59,60]. MYD88 is a significant target for innovative therapies in pancreatic cancer due to its crucial molecular role in linking various upstream ligand–receptor complexes [61]. We evaluated the expression level of MYD88 in 179 primary tumor tissues and 171 normal tissues from the TCGA database. Our findings revealed a significantly higher expression of MYD88 in cancer tissues compared to normal tissues (*p* < 0.001, Figure 4C). Furthermore, Kaplan–Meier survival analyses identified a significant association between MYD88 expression and overall survival in PDAC patients from the TCGA cohort (HR = 1.58, *p* = 0.045, Supplementary Figure S3). Finally, we validated the higher mRNA expression of MYD88 in Gem-R PDAC cell lines compared to parental cell lines through qRT-PCR assays (BxPC-3 cell line: *p* < 0.05, Figure 4D and *p* < 0.05, Figure 4D). Consequently, our findings suggest that the combination of ABE and Gem could potentially target the MYD88/NF-κB signaling pathway.

### 2.5. Combined Treatment with Gemcitabine and ABE Downregulates P-gp through the MYD88/TLR3/NF-κB Signaling Pathway

**Figure 5:**
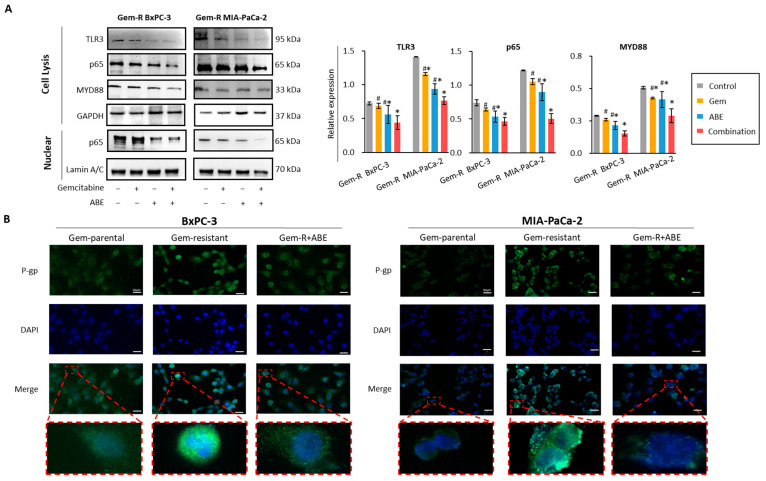
The combination treatment of Gem and ABE has been shown to regulate the MYD88/TLR3/NF-κB/P-gp complex in Gem-R PDAC cell lines. (**A**) Western blot analysis was performed on Gem-R PDAC cells treated with Gem, ABE, and their combination for 48 h. The relative levels of TLR3, MYD88, and p65 were quantitatively analyzed by comparing them to the control GAPDH expression. (**B**) Representative images for an immunofluorescence assay were obtained to evaluate P-gp expression in parental PDAC cell lines, Gem-R PDAC cell lines, and Gem-R PDAC cell lines treated with ABE for 48 h. * *p* indicates *p* < 0.05 vs. control group; # *p* indicates *p* < 0.05 vs. combination group.

The Toll-like receptor (TLR) family, which includes *TLR1-10*, comprises transmembrane glycoproteins primarily expressed in immune cells. In particular, *TLR3* and *TLR4* can activate transcription factor NF-κB through both MYD88-dependent pathways and MYD88-independent pathways [62]. To investigate whether the MYD88/TLR3/NF-κB signaling pathway is a key target pathway of the combined treatment of Gem and ABE, WB was performed to assess the expression levels of key genes involved in the MYD88/TLR3/NF-κB axis when treated with Gem, ABE, and their combination. Our results revealed that TLR3, MYD88, and p65 were significantly downregulated by the combined treatment in both Gem-R BxPC-3 and Gem-R MIA-PaCa-2 cell lines (Figure 5A). In addition, the MYD88/NF-κB signaling pathway is linked to chemoresistance, as it has the ability to upregulate efflux transporters such as P-gp [56,63]. To evaluate the protein expression level of P-gp, we conducted immunofluorescence analysis (excitation: 488 nm; emission: 564 nm). Compared to the parental PDAC cell lines, the expression of P-gp in the Gem-R cell lines was significantly higher, and the treatment with ABE reduced the expression of P-gp in both PDAC cell lines after treatment (Figure 5B).

### 2.6. The Combination of Gemcitabine and ABE Suppressed the Growth of PDOs

**Figure 6:**
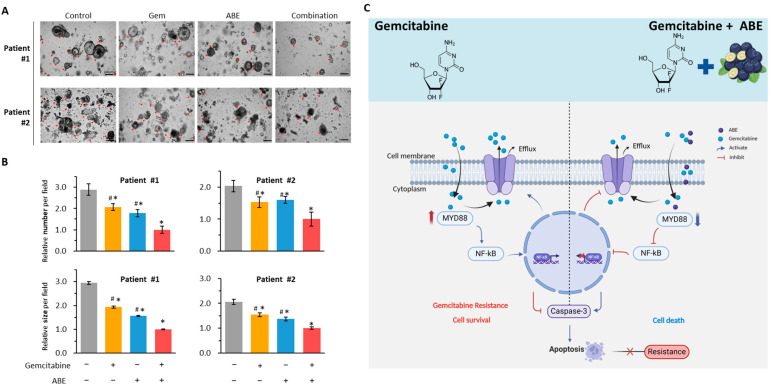
The combination of Gem and ABE demonstrates effective anticancer activity in PDAC patient-derived 3D organoid (PDO) models. (**A**) Representative images of PDOs treated with Gem, ABE, and their combination for 48 h. (**B**) The average number and size of PDOs treated with Gem, ABE, and their combination for 48 h. (**C**) A schematic illustration of ABE-induced reversal of Gem resistance in PDAC. This illustration reveals that Gem can activate the MYD88/NF-Κb axis, resulting in chemoresistance by upregulating the efflux transporters (P-gp) and hindering cellular apoptosis in PDAC cells (**left**). In contrast, ABE helps counteract chemoresistance by downregulating MYD88 and downstream signaling pathways in PDAC cells (**right**). * *p* indicates *p* < 0.05 vs. control group; # *p* indicates *p* < 0.05 vs. combination group.

Three-dimensional organoid culture systems have emerged as valuable tools for modeling various aspects of cancer biology, particularly in investigating drug responses and evaluating treatment efficacy. In this study, we used tumor organoid models derived from two patients to examine the anti-tumor effects of Gem and ABE. As anticipated, our results demonstrated that the combination of Gem and ABE significantly suppressed both the size and number of organoids (Figure 6A). In terms of the number of organoids, when compared to individual treatments (Patient1: combination vs. Gem: FC = 0.48, *p* < 0.05; combination vs. ABE: FC = 0.56, *p* < 0.05, Figure 6B; Patient2: combination vs. Gem: FC = 0.65, *p* < 0.05; combination vs. ABE, FC = 0.62, *p* <0.05; Figure 6B), the combination was more effective. Similarly, the combination of Gem and ABE significantly reduced organoid sizes compared to individual treatments (Patient1: combination vs. Gem: FC = 0.52, *p* < 0.05; combination vs. ABE: FC = 0.64, *p* < 0.05, Figure 6C; Patient2: combination vs. Gem: FC = 0.65, *p* < 0.05; combination vs. ABE, FC = 0.73, *p* <0.05; Figure 6C). These findings support the notion that the combination of Gem and ABE effectively inhibit the growth of PDOs, corroborating the results of our cell culture experiments.

## 3. Discussion

Since the FDA’s approval in 1996, Gem-based chemotherapy has been used extensively as a standard treatment regimen in multiple kinds of solid cancers, including ovarian, breast, and non-small cell lung cancer [64]. Combination chemotherapeutic strategies incorporating Gem remain the preferred treatment for advanced PDAC [65]. However, chemotherapeutic resistance often occurs in PDAC patients. In particular, the development of resistance to Gem presents an important clinical challenge in the effective therapeutic management of patients with PDAC [66]. Several studies have demonstrated the significant role of ABC transporters like P-gp, BCRP, and MRP1 in mediating resistance to Gem chemotherapy [12], and the ability of ABC transporter inhibitors to sensitize tumor cells to chemotherapeutic agents has been researched extensively. While some positive outcomes have been observed in preclinical studies and early-phase clinical trials, no effective MDR-reversing agents targeting ABC transporters have been approved for clinical use thus far [67].

As such, natural products have been explored for their potential anticancer activity and low toxicity in healthy tissues and have recently been reported to overcome drug resistance [68,69]. In our study, we aimed to combine Gem with ABE to investigate whether ABE could enhance the sensitivity of Gem-R cells to Gem. Following the administration of a combination of Gem and ABE to Gem-R cells, our results revealed that ABE synergistically increased the sensitivity of Gem-R cells to Gem. Moreover, the combination of ABE and Gem effectively inhibited cell viability, clonogenicity, migration, and invasion while inducing a higher apoptosis rate in PDAC cells. We further found that the combined ABE and Gem treatment could significantly strengthen the anticancer activity in inhibiting PDAC PDOs, supporting our cell culture-based findings.

To investigate the mechanism by which ABE enhances sensitivity to Gem, we analyzed gene expression profiles from two publicly available GEO datasets and compared parental and Gem-R PDAC cells. Our analysis revealed that MYD88, an adaptor molecule for Toll-like receptors [70,71,72,73], was upregulated in Gem-R PDAC cells. Furthermore, we found that TLR3 can activate the transcription factor NF-κB through both the MYD88-dependent pathway and the MYD88-independent pathway [62]. The MYD88/NF-κB signaling pathway is linked to chemoresistance as it can elevate the expression of efflux transporters such as P-gp [56,74,75]. In the current study, we revealed that MYD88 is upregulated in tumor tissues and that high expression levels of MYD88 correlate with poor prognosis in PDAC. Second, we discovered that MYD88 is also upregulated in Gem-R cell lines. Therefore, we hypothesized that ABE could target MYD88, potentially restoring sensitivity to Gem in Gem-R cell lines. Our WB results showed that co-treatment of Gem and ABE inhibited the expression of MYD88, TRL3, and P65 expression. These findings provide further evidence for MYD88/TRL3/NF-κB as a critical pathway in the ability of ABE to overcome Gem resistance. However, the present study lacks definitive evidence to establish whether alterations in TLR3 expression are MYD88-dependent. Additionally, the co-treatment of Gem and ABE suppressed the expression of P-gp, as shown by immunofluorescence staining. These findings provide compelling evidence for the involvement of the MYD88/TRL3/NF-κB/P-gp axis as the target mechanism through which ABE counteracts Gem resistance in PDAC cells (Figure 6C).

Finally, we confirmed the anti-tumor efficacy of Gem and ABE by utilizing tumor organoid models derived from two PDAC patients. However, we did not generate the Gem-R organoids, which may limit our finding validation in vivo. Based on these, we propose that we can validate these findings in animal models in the future.

In summary, our study provides valuable insights into the potential of ABE to overcome Gem resistance in PDAC. Our data reveal a crucial mechanism underlying the synergistic interaction between ABE and Gem. These findings contribute to the growing body of evidence supporting the potential of ABE as a novel therapy for PDAC, particularly for patients confronting Gem resistance.

## 4. Materials and Methods

### 4.1. Cell Culture

The PDAC cell lines BxPC-3 and MIA-PaCa-2 were sourced from the American Type Culture Collection (ATCC, Manassas, VA, USA). The cell lines were cultured in RPMI medium (Gibco, Carlsbad, CA, USA) with 1% penicillin/streptomycin (Sigma-Aldrich, St. Louis, MO, USA) and 10% fetal bovine serum (Gibco, Waltham, MA, USA). The cells were cultured at 37 °C with 5% CO2 in a humidified environment. The adherent cells were harvested using 0.05% trypsin (Invitrogen, Carlsbad, CA, USA). Gem-R MIA-PaCa-2 and Gem-R BxPC-3 were established by continuously culturing the cells with increasing doses of Gem, as described previously [3].

### 4.2. Herbal Preparations

The aronia berry extract utilized in this study (Aronia Berry Complex, EuroPharma USA, Green Bay, WI, USA) was a powdered product characterized by colors ranging from purple to dark red. It was derived from the fruit of the *Aronia Melanocarpa* plant, extracted in 70% ethanol, and standardized to a 40% polyphenol content. The extract was initially dissolved in dimethyl sulfoxide to create stock concentrations and then further diluted to suitable levels in the culture media.

### 4.3. Reagents

Gemcitabine (Sigma-Aldrich, St. Louis, MO, USA) was dissolved in dimethyl sulfoxide (DMSO, Sigma-Aldrich). The stock solutions of Gem (10 μM) and ABE (100 mg/mL) were carefully stored at −20 °C in the dark, ensuring their stability and reliability. These stock solutions were diluted with a complete culture medium to the necessary experimental concentrations before each application.

### 4.4. Cell Counting Kit-8 Assays

For the Cell Counting Kit-8 (CCK-8) assay, a CCK-8 kit (Dojindo, Kumamoto, Japan) was used to measure cell viability according to the instructions as follows: Initially, to confirm the Gem-resistant properties, the proliferation rates were compared between Gem-R and their parental counterparts by subjecting them to escalating doses of Gem. Cells were seeded in 96-well flat plates at a density of 5 × 10^3^ cells per well and incubated for 24 h. Thereafter, cells were treated with increasing concentrations of Gem (0–1200 nM). After 48 h of treatment, 10 μL of CCK-8 solution was added to each well and incubated for 2 h. The absorbance of the product was measured at a wavelength of 450 nm (OD450) using a microplate reader from Tecan Trading AG (Tecan Trading AG, Männedorf, Switzerland).

Subsequently, to investigate the influence of ABE and Gem combination on PDAC cell proliferation, cells were seeded in 96-well plates at a density of 2 × 10^3^ cells/well in 100 µL of complete culture medium and incubated for 24 h. Subsequently, the cells were exposed to appropriate concentrations of Gem (800 nM), ABE (90 μg/mL), and their combination. The cell proliferation rates were evaluated at different time points.

### 4.5. Drug Response Testing

The Gem-R PDAC cell lines were plated at a density of 5 × 10^3^ cells per well in 96-well plates and incubated for 24 h before drug administration. Subsequently, the cells were exposed to varying concentrations of Gem (0, 400, 800, and 1200 nM), ABE (0, 30, 60, 90, and 120 μg/mL), and twenty different combinations of these drugs for a duration of 48 h, to identify the synergistic concentrations. Cell viability was assessed using the CCK8 assay. Synergy scores were subsequently calculated using SynergyFinder 3.0, a freely accessible tool specifically designed for interactive analysis and visualization of combination response outcomes [76].

### 4.6. Colony Formation Assay

For the cell colony formation assay, 5 × 10^2^ cells were seeded per well in 6-well flat plates and then treated with Gem, ABE, and their combination for 48 h. Following this treatment, colony formation was allowed to proceed for a duration of 7 to 10 days, with the culture medium refreshed every three days. At the end of this incubation period, the cell colonies were fixed with 4% paraformaldehyde in PBS (Thermo Fisher Scientific, Fair Lawn, NJ, USA) for 30 min and stained with 1% crystal violet (Thermo Fisher Scientific). The number of colonies was quantified using ImageJ 1.53q software.

### 4.7. Wound Healing Assay

In the cell wound healing assay, we seeded 5 × 10^5^ cells per well in 6-well flat plates following a 48 h treatment with Gem, ABE, or their combination. After the cells reached 80% to 90% confluence, we utilized a sterile 20 μL micropipette tip to create a controlled scratch in the monolayer. This was followed by a wash with a serum-free medium to eliminate detached cells. Subsequently, the cells were cultured in a complete medium. Photographs of the cells were captured 24 h after the formation of the wound, and the percentage of wound closures was determined using ImageJ software.

### 4.8. Invasion Assays

To conduct cell invasion assays, BioCoat Matrigel Invasion Chambers with 8.0 μm Pore Polyester Membrane (BD Biosciences, Franklin Lakes, NJ, USA) were utilized. For the invasion assay, 5 × 10^4^ cells per well were seeded in 24-well flat plates after treatment with Gem, ABE, and their combination for 48 h. The cells were then transferred onto inserts in serum-free medium and subsequently moved to wells containing culture medium supplemented with 10% FBS. After a 48 h incubation period, the cells that had invaded the bottom surface of the membrane were fixed and stained using a Diff-Quick staining kit (Thermo Fisher Scientific). The stained cells were later quantified under a microscope.

### 4.9. Apoptosis Assay

For apoptosis assays, Muse™ Annexin V and Dead Cell kits (Luminex Corp, Austin, TX, USA) were utilized following the manufacturer’s guidelines. In these experiments, 5 × 10^5^ cells were seeded per well in 6-well flat plates. Following treatment with Gem, ABE, and their combination for 48 h, the cells were harvested. Subsequently, 100 μL of cell suspension was mixed with 100 μL of Muse Annexin V & Dead Cell Reagent. The percentage of apoptotic cells was assessed using a Muse™ Cell Analyzer (Millipore Corp, Billerica, MA, USA) in accordance with the manufacturer’s instructions.

### 4.10. Gene Enrichment and Pathway Analysis

To identify differentially expressed genes in Gem-R PDAC cells, gene expression profiles from two publicly available datasets (GSE148200 and GSE140077) sourced from the Gene Expression Omnibus (GEO) database (https://www.ncbi.nlm.nih.gov/geo/, accessed on 28 August 2022) were analyzed. Differential gene expression across various groups was examined using the “DEseq2” package in R, with a significance threshold of *p* < 0.05 and a Log_2_FC (Logarithm of Fold Change) exceeding ±0.5.

The Kyoto Encyclopedia of Genes and Genomes (KEGG) pathway analysis was performed using the DAVID bioinformatics database (https://david.ncifcrf.gov/, accessed on 28 August 2022). The KEGG pathway enrichment analysis was visualized using the “ggplot2” package in R.

### 4.11. Isolation of Cytosolic and Nuclear Extracts

After treatment with Gem, ABE, and their combination for 48 h, cytosolic and nuclear extracts were isolated from cells using a nuclear extraction kit (Active Motif, Carlsbad, CA, USA) according to the manufacturer’s instructions. These extracts were collected and stored in aliquots at −80 °C.

### 4.12. Protein Isolation and Western Blot

Total protein was extracted from PDAC cell lines treated for 48 h with Gem, ABE, or their combination. The cells were harvested using a plastic scraper. Subsequently, the cells were lysed with ice-cold protein extraction solution RIPA containing a protease inhibitor cocktail (Thermo Fisher Scientific). Protein concentration was determined using the BCA procedure (Thermo Fisher Scientific). Equal amounts of protein samples were separated via SDS-PAGE using 6% or 10% Mini-PROTEAN TGXTM Precast Gels (BIO-RAD, Hercules, CA, USA) and then transferred onto nitrocellulose membranes, followed by an additional transfer onto a 0.45 μm PVDF membrane (Cytiva, Marlborough, MA, USA). The membranes were blocked with 5% bovine serum albumin in Tris-buffered saline with 0.1% Tween-20 for 1 h at room temperature. Subsequently, the membranes were incubated with primary antibodies, including anti-Bax (1:1000, 5023S; Cell Signaling Technology [CST], Danvers, MA, USA), anti-Cleaved Caspase 9 (1:1000, 9505; CST), anti-PARP (1:1000, 9532S; CST), anti-TLR3(1:1000, PA5-20183; Thermo Fisher Scientific), anti-MYD88 (1:1000, 23230-1-AP; Proteintech, Rosemont, IL, USA), anti-P65 (1:1000, 3034; CST), anti-Lamin A/C (1:2000, 10298-1-AP, Proteintech), anti-Vimentin (1:2000, 10366-1-AP, Proteintech), anti-MMP-9 (1:1000, 10375-2-AP, Proteintech), anti-E-cadherin (1:2000, 10375-2-AP, Proteintech), and anti-GAPDH (1:2000, 5174T; CST) at 4 °C overnight. After three washes with TBST, the membranes were incubated with corresponding anti-rabbit (1:2000, 7074; CST) or anti-mouse (1:2000, 7076; CST) secondary antibodies for 1 h at room temperature. The blot was visualized using an HRP-based chemiluminescence kit (Thermo Fisher Scientific) with Gel Imaging Systems (BIO-RAD). GAPDH protein served as an internal control, and the intensities of the protein bands were quantified using ImageJ software.

### 4.13. Quantitative Reverse Transcription PCR (qRT-PCR)

Total RNA extraction was performed using the Qiagen miRNeasy Kit (Qiagen, Hilden, Germany). Subsequently, cDNA was synthesized by reverse transcription of total RNA using the High-Capacity cDNA Reverse Transcription Kit from Thermo Fisher Scientific. RT-PCR assays were conducted with the QuantStudio 6 Flex RT-PCR System (Applied Biosystems, Foster City, CA, USA), following the manufacturer’s instructions and utilizing the SensiFAST SYBR Lo-ROX Kit from Bioline (London, UK). The relative mRNA expression levels of MYD88 were determined using the 2^−ΔΔCt^ method and normalized to the expression of *β-actin*, acting as the internal control. The primer sequences can be found in Supplementary Table S1.

### 4.14. Immunofluorescence Assay

For the immunofluorescence assay, 5 × 10^5^ cells per well were seeded in 6 cm flat plates with slides. Following an 18-h incubation period, the cells were treated with Gem, ABE, or their combination for 48 h. The slides were subsequently fixed with 4% paraformaldehyde for 10 min at room temperature, then permeabilized with 0.5% Triton-X-100 (Thermo Fisher Scientific) for 10 min at room temperature. To remove endogenous peroxides, the slides were treated and subsequently blocked in TBST (Tris-buffered saline with Tween-20) containing 3% BSA for 1 h at room temperature. The slides were then incubated with an anti-P-glycoprotein Polyclonal antibody (1:500, 22336-1-AP, Proteintech) overnight at 4 °C, followed by incubation with a FITC-labeled secondary antibody (1:1000, A-21202; Thermo Fischer Scientific) for 1 h at 37 °C. Following incubation, the slides were subjected to three additional washes with phosphate-buffered saline, each lasting five minutes. Finally, the cell nucleus was stained with DAPI (Thermo Fischer Scientific), and all images were captured using a Carl Zeiss fluorescent microscope (Zeiss, Oberkochen, Germany).

### 4.15. Patient-Derived 3-Dimensional Tumor Organoids (PDOs)

PDOs from PDAC patients were generated as described in a previous study [77]. With approval from the institution’s ethics committees, written informed consent was obtained from all patients. To ensure confidentiality and anonymity, patients were coded following ethical guidelines outlined in the Declaration of Helsinki. For the human organoids, the Human Complete Feeding Medium (hCPLT) consisted of PancreaCultTM Organoid Growth Medium (STEMCELL Technologies, Cambridge, MA, USA), supplemented with EGF (STEMCELL Technologies) and prostaglandin E2 (STEMCELL Technologies), following the manufacturer’s instructions. PDAC organoids were seeded in a 24-well plate to form a dome in 40 μL Matrigel (Corning, Tehama County, CA, USA) with 500 μL of hCPLT. The domes were then divided into 4 groups and appropriate concentrations of Gem (800 nM), ABE (90 μg/mL), and their combination. After 7 days of treatment, the number and size of the organoids were analyzed through microscopy (magnification ×100) and measured using ImageJ software.

### 4.16. Statistical Analysis

Statistical analysis was conducted utilizing SPSS software version 21.0 and GraphPad Prism version 6.0. Student’s *t*-test was employed to assess the significance of observed differences between the two groups, while one-way analysis of variance (ANOVA) was utilized to evaluate differences among multiple comparisons. All experiments were performed in triplicate on independent biological replicates, and the data were presented as mean ± standard deviation. A significance level of *p* < 0.05 was considered statistically significant.

## 5. Conclusions

We firstly provided a novel insight into the potential of ABE to overcome Gem-R in PDAC cells using a systematic series of Gem-resistant cell culture and patient-derived tumor organoids experiments. Additionally, we observed that the MYD88/NF-κB/P-gp axis plays a crucial role in Gem resistance in PDAC, and ABE could overcome this resistance by downregulating MYD88 and its downstream signaling pathways in PDAC cells (Figure 6C), suggesting ABE as a promising and cost-effective approach to enhance therapeutic outcomes in PDAC. These results warrant further exploration of ABE in clinical settings to validate its potential as an adjunct therapy for improving treatment efficacy in pancreatic cancer patients. In the next phase of our research, we should further investigate the optimal dose of ABE to effectively counter Gem resistance at the minimum required dosage.

## Data Availability

Data are contained within the article.

## Supplementary Materials

The following supporting information can be downloaded at: https://www.mdpi.com/article/10.3390/ph17070911/s1, Supplementary Figure S1: The cell viability of Gem-R BxPC-3 (A) and Mia PaCa-2/Gem-R (B) cells treated with different concentrations of ABE for 48 h. Supplementary Figure S2: Western blot analysis was performed on Gem-R PDAC cells treated with Gem, ABE, and their combination for 48 h. The relative levels of E-cadherin, Vimentin, and MMP9 were quantitatively analyzed by comparing them to the control GAPDH expression. Supplementary Figure S3: Kaplan–Meier survival curves of the selected genes, MYD88, were analyzed by PDAC cases from the TCGA public database. Supplementary Figure S4: Original Western blots. Supplementary Table S1: Primer sequences and their PCR conditions in this study.
