# Supplementary material for: Aronia Berry Extract Modulates MYD88/NF-kB/P-Glycoprotein Axis to Overcome Gemcitabine Resistance in Pancreatic Cancer
Source: Pharmaceuticals (Basel). 2024 Jul 9;17(7):911. doi: 10.3390/ph17070911 (PMC11279572; doi:10.3390/ph17070911)
Supplement: Supplementary file 1 [file pharmaceuticals-17-00911-s001.zip › pharmaceuticals-3074955-supplementary.pdf]

Supplementary Figure S1:

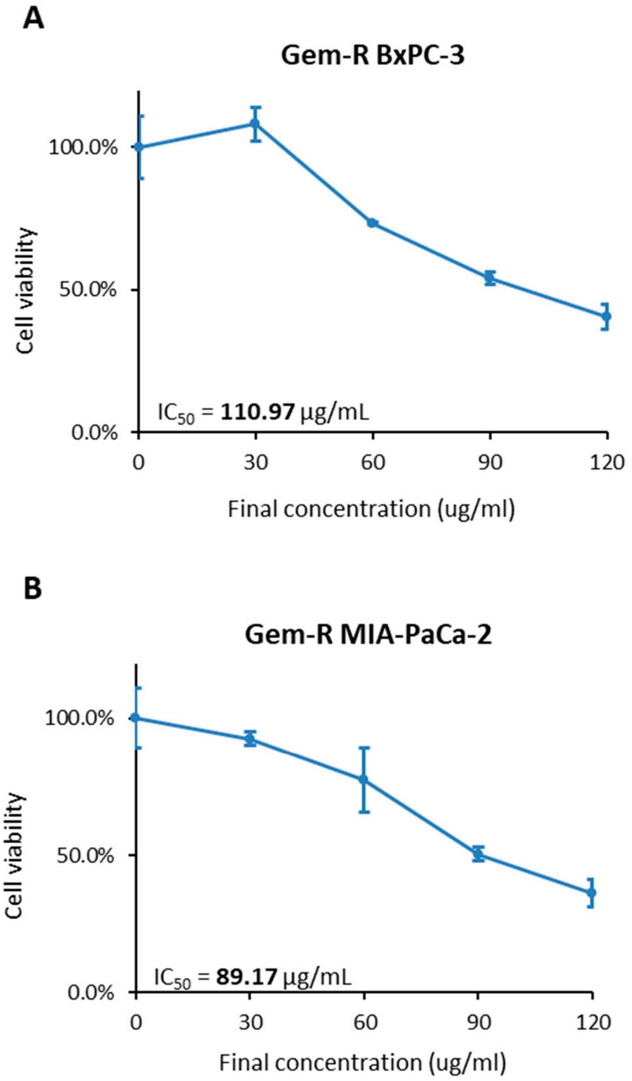

Supplementary Figure S2:

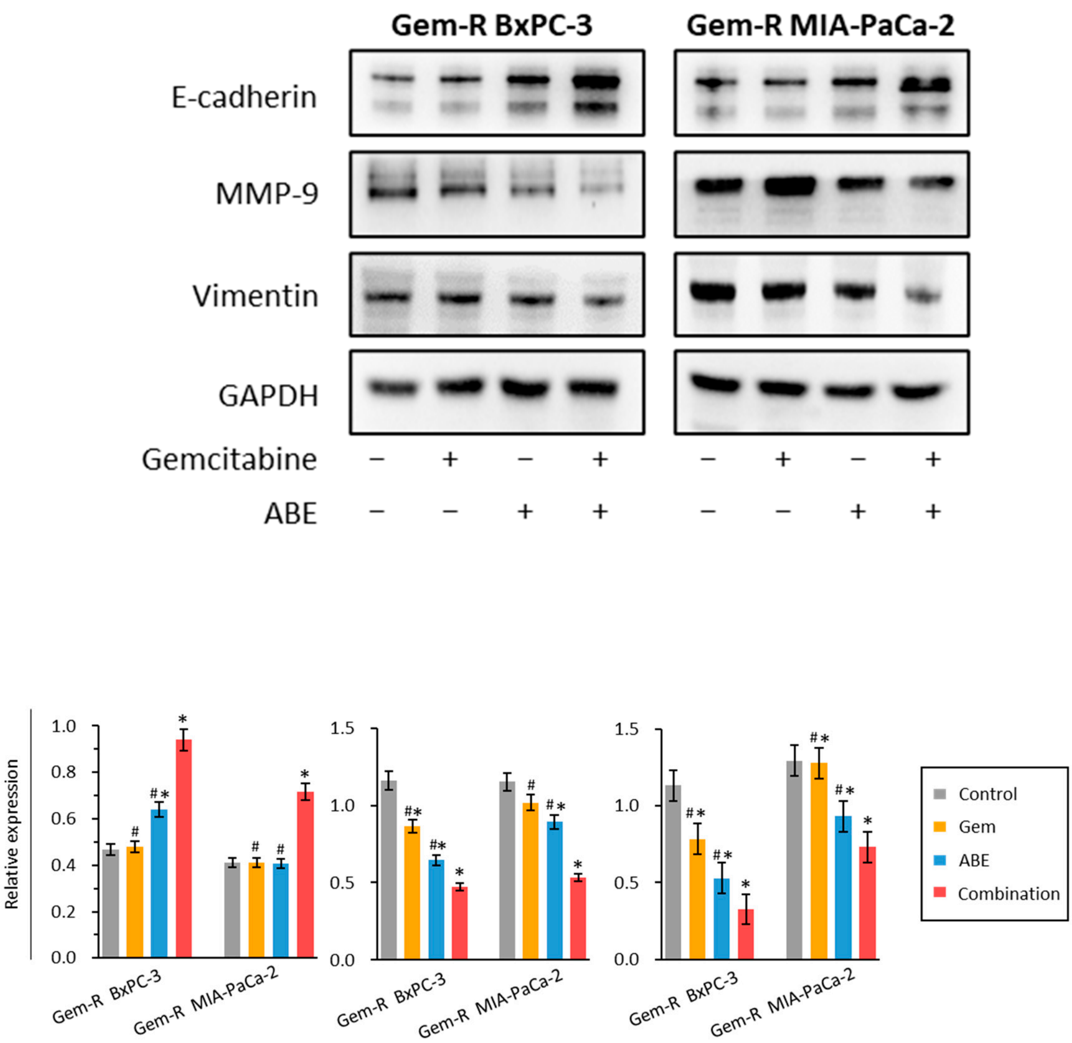

Supplementary Figure S3:

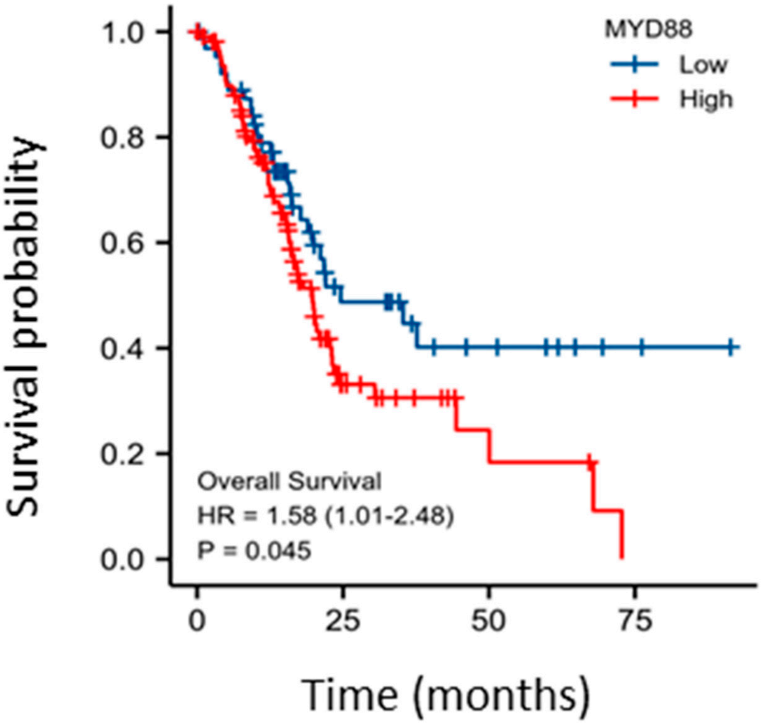

Supplementary Figure S4: Original western blots

Figure 3:

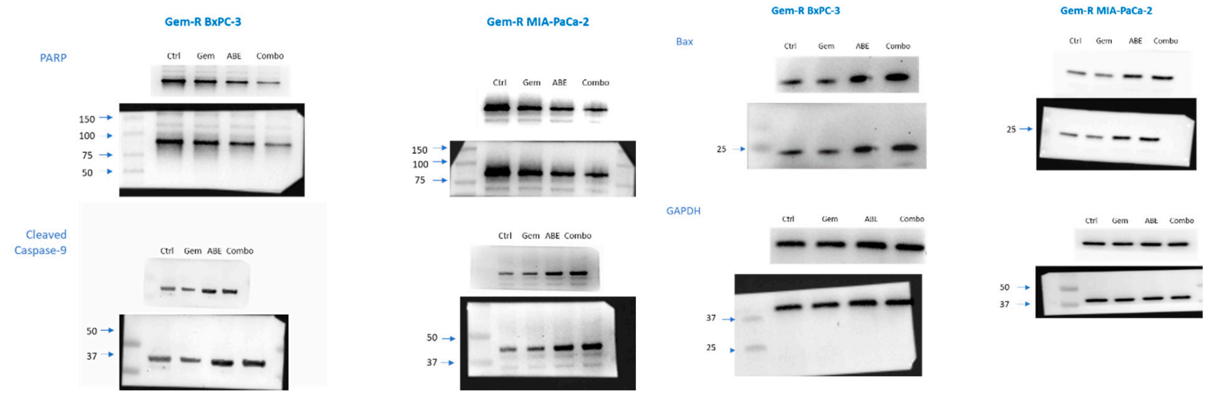

Figure 5

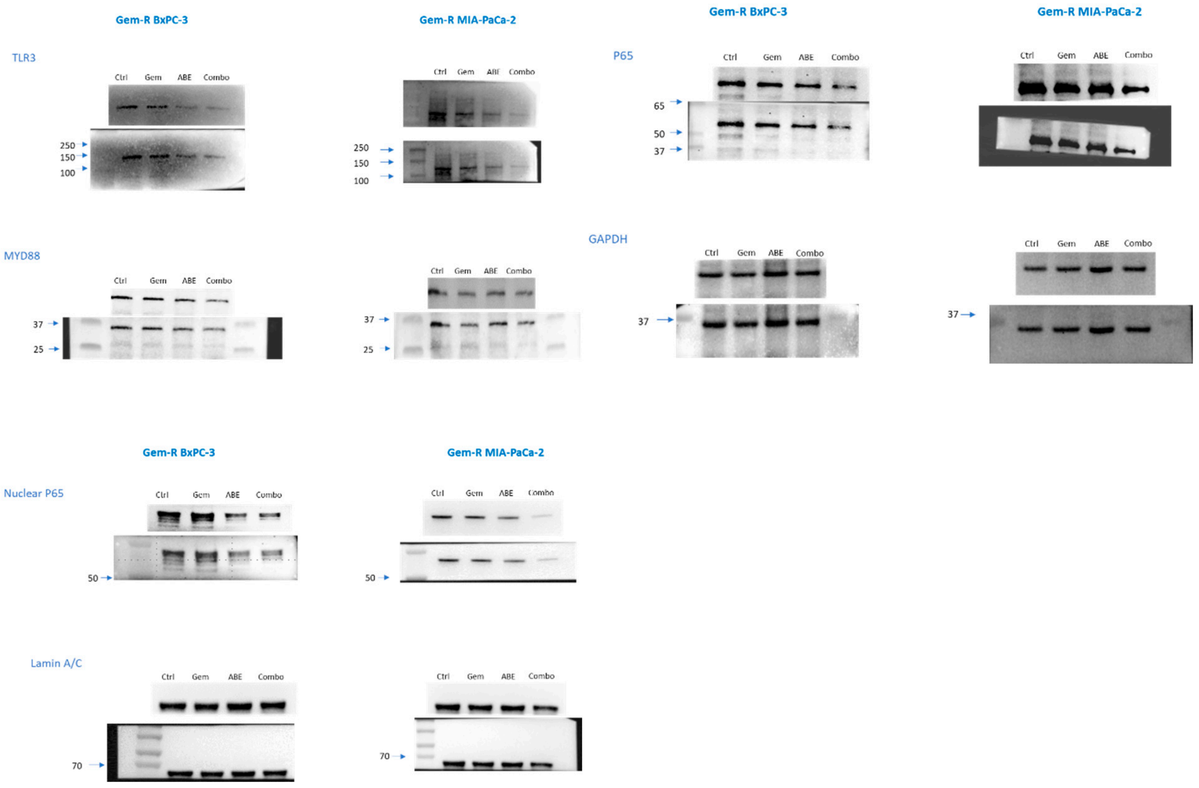

Supplementary Figure S2

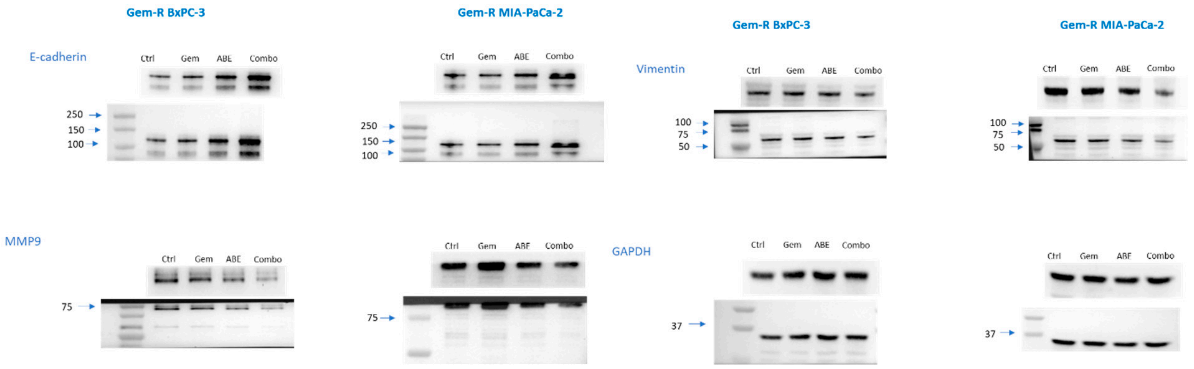

Ctrl: Control group

Gem: Gemcitabine Group

ABE: Aronia berry group

Combo: Combination group of Gem and ABE

Supplement Table 1: Primer sequences for qPCR assays

Gene name	Forward (5’-3’)	Reverse (5’-3’)
Actin	CCTTTGCCGATCCGCCG	GATATCATCATCCATGGTGAGCTG
MYD88	GGCTGCTCTCAACATGCGA	CTGTGTCCGCACGTTCAAGA
